# Genome-wide identification and expression analysis of the WRKY transcription factors related to sesquiterpenes biosynthesis in *Atractylodes lancea*

**DOI:** 10.3389/fgene.2025.1551991

**Published:** 2025-05-15

**Authors:** Hua Liang, Weiwei Liu, Zhiqiang Zhao, Yaqian Li, Liangping Zha

**Affiliations:** ^1^ College of Pharmacy, Anhui University of Chinese Medicine, Hefei, China; ^2^ Institute of Conservation and Development of Traditional Chinese Medicine Resources, Anhui Academy of Chinese Medicine, Hefei, China; ^3^ MOE-Anhui Joint Collaborative Innovation Center for Quality Improvement of Anhui Genuine Chinese Medicinal Materials, Anhui University of Chinese Medicine, Hefei, China

**Keywords:** *Atractylodes lancea*, WRKY transcription factors, genome-wide analysis, expression patterns, sesquiterpenes

## Abstract

**Introduction:**

*Atractylodes lancea* (Thunb.) DC., a widely utilized herb in traditional Chinese medicine, contains sesquiterpenoids and polyacetylenes as its primary bioactive components. The WRKY gene family plays a critical role in regulating various biological processes in plants. However, the molecular mechanism underlying AlWRKY regulation of terpenoid synthesis unclear.

**Methods:**

The *AlWRKY* gene family members were identified through bioinformatics approaches. Gene structures, motifs, and phylogenetic relationships were analyzed. Subsequently, their expression profiles across different geographical origins were investigated using transcriptome data. Furthermore, preliminary validation was performed via methyl jasmonate treatment and molecular docking, with a focus on the *AlWRKY20* and *AlWRKY37* genes.

**Results:**

In this study, 65 *AlWRKY* genes with conserved domains were identified in *A. lancea* and classified into three groups: Group I (17 members), Group II (33 members), and Group III (15 members). Tissue-specific expression profiling revealed five rhizome-enriched *AlWRKY* genes (*AlWRKY13*, *AlWRKY20*, *AlWRKY21*, *AlWRKY37*, and *AlWRKY49*) were highly expressed in Hubei accessions compared to Jiangsu accessions, and co-expression analysis demonstrated their strong correlation with 16 *AlTPS* genes. Quantitative PCR (qPCR) validation confirmed the specific upregulation of *AlWRKY20*, *AlWRKY21*, *AlWRKY37*, and *AlWRKY49* in Hubei rhizomes, consistent with the accumulation patterns of sesquiterpenes (hinesol, γ-eudesmol, and elemol). Methyl jasmonate (MeJA) induction experiments (12 h) revealed coordinated upregulation of *AlWRKY20*, *AlWRKY37*, *AlTPS70*, *AlTPS71*, concomitant with significantly increased cis-β-farnesene and α-curcumene content. Molecular docking analysis revealed strong binding affinities of AlWRKY20 to the AlTPS70/AlTPS71 promoter and of AlWRKY37 to the AlTPS70 promoter. Subcellular localization analysis demonstrated that both AlWRKY20 and AlWRKY37 are localized in the nucleus. These results suggest that AlWRKY20 and AlWRKY37 likely function as regulators of sesquiterpene biosynthesis, positively regulating cis-β-farnesene and α-curcumene production through *AlTPS* gene modulation.

**Discussion:**

This study lays the groundwork for further exploration of the molecular mechanisms and functional validation of WRKY transcription factors in *A. lancea*.

## 1 Introduction

*Atractylodes lancea* (Thunb.) DC., a member of the Asteraceae family, constitutes a primary source of the traditional Chinese medicine *Atractylodis*, commonly referred to as “Cangzhu” in China. Dried rhizomes of this plant have been utilized for treating various diseases, such as spleen deficiency syndrome (SDS), across China, Japan, South Korea, and North Korea over an extended period (Peng et al., 2012; Xue et al., 2018). *A. lancea* contains volatile oils containing sesquiterpenes, terpenoids, polyacetylenes, monoterpenes, and steroids (Liu et al., 2016; Xu et al., 2016; Zhang et al., 2021). These components have garnered increasing scholarly interest in recent years. Prior research has identified hinesol, β-eudesmol, atractylon, and atractylodin as the main active components of the volatile oil components in *A. lancea* (Zhang et al., 2010; Tshering et al., 2021). Nevertheless, the composition of volatile oil in *A. lancea* varies across geographical regions. For example, the content of hinesol and β-eudesmol in Hubei significantly exceeds that in Jiangsu (Guo et al., 2008; Tsusaka et al., 2019; Xu et al., 2016; Zhang et al., 2023). This variation may correlate with gene regulation within terpenoid synthesis pathways (Zhang et al., 2023; Zhang et al., 2024).

Previous studies, including our own, have identified multiple genes associated with terpenoid synthesis in *A. lancea*. The *AlAACT* gene (Wu et al., 2022), along with *AlDXS* and *AlDXR* genes, were cloned in *A. lancea* and expressed in a prokaryotic system (Xu R. et al., 2023). Notably, *AlSQS1* and *AlSQS2* encode functional enzymes that catalyze the conversion of two farnesyl pyrophosphate molecules into squalene (Wu et al., 2021). Similarly, *AlTPS1* and *AlTPS2* utilize farnesyl pyrophosphate as a substrate to synthesize the sesquiterpenoids elemol and β-farnesene, respectively (Wu et al., 2023). Although significant progress has been made in identifying functional genes involved in terpenoid biosynthesis in *A. lancea*, limited research has been conducted on its transcription factors. In our preliminary transcriptome analysis of *A. lancea* rhizomes, *AlWRKY* genes exhibited co-expression patterns with *AlTPS* genes and correlated strongly with sesquiterpenoid content, prompting further investigation.

WRKY transcription factors (TFs) significantly contribute to the regulation of secondary metabolism in various medicinal plants. Recent studies have increasingly focused on the role of WRKY TFs in regulating terpenoid biosynthesis (Wang et al., 2021; Sun et al., 2018; Goyal et al., 2023). In *Litsea cubeba*, *LcWRKY17* interacts with the W-box in the *LcTPS42* promoter, and its overexpression markedly enhances monoterpene synthesis (Gao et al., 2023). Similarly, the *PqWRKY1* transcription factor plays a pivotal role in regulating triterpene ginsenoside biosynthesis in *Panax quinquefolius* (Sun et al., 2013). In *Artemisia annaua*, *WRKY1* (*AaWRKY1*) has been identified as a key regulator of amorpha-11-diene synthesis during terpene biosynthesis (Ma et al., 2009). Additionally, WRKY TFs involved in terpenoid biosynthesis have been identified in several medicinal plants, including *Phyllostachys edulis* (Zhang Z. J. et al., 2022), *Carthamus tinctorius* L (Li et al., 2020), *Medicago sativa* L (Li et al., 2023). A total of 86 HpWRKY and 63 AkWRKYS TFs have been identified in *Hypericum perforatum* and *Akebia trifoliata* (Zhou et al., 2022; Zhu et al., 2022). Similarly, 77 WRKY and 72 WRKY members have been identified in *Scutellaria baicalensis* Georgi and *Taraxacum kok*-*saghyz* Rodin genome (Zhang C. J. et al., 2022; Cheng et al., 2022). However, the whole-genome characterization of this gene family in *A. lancea* remains unexplored. Identifying *WRKY* genes in *A. lancea* will provide insights into the genetic mechanisms underlying its local adaptation and medicinal compound biosynthesis, thereby linking genomic variation with metabolomic diversity in this economically important medicinal species.

Although numerous *WRKY* genes have been functionally characterized in other species, a comprehensive analysis of the WRKY gene family in *A. lancea* is still lacking. In this study, 65 members of the WRKY gene family, designated AlWRKY, were identified in *A. lancea*. A systematic bioinformatics analysis was conducted, encompassing the phylogenetic relationships of AlWRKY proteins, conserved domain motifs, cis-elements and collinearity. Additionally, expression patterns of *AlWRKY* genes across various tissues and andmethyl jasmonate (MeJA) treatments were examined, followed by molecular docking analyses to assess AlWRKY binding potential with AlTPSs promoters. Collectively, this comprehensive study not only contribute to elucidating the mechanistic role of *AlWRKY* genes in modulating terpenoid biosynthesis pathways in *A. lancea,* but also opens avenues for metabolic engineering and sustainable harvesting strategies.

## 2 Materials and methods

### 2.1 Plant materials and treatment

Fresh plant tissues from 3-year-old wild *A. lancea* were collected from Yingshan (Hubei Province, China) and Nanjing (Jiangsu Province, China) for genome and transcriptome sequencing. Tissues were separated into roots, stems, and leaves, with three biological replicates per organ. These samples were immediately frozen in liquid nitrogen and stored at −80°C. Meanwhile, 3-month-old seedlings from Yingshan (Hubei, China) were treated with 200 μM methyl jasmonate (MeJA) for 0 h, 6 h, 12 h, and 24 h. Three replicates were performed for each treatment for quantitative real-time PCR (qPCR) and volatile chemical component analysis.

### 2.2 Identification and sequence analysis of *WRKY* genes in *A. lancea*

The hidden Markov model (HMM) file corresponding to the WRKY domain (PF03106) was downloaded from the Pfam protein family database (https://www.ebi.ac.uk/interpro/entry/pfam/#table, accessed 20 October 2023). HMMER 3.0 software (http://hmmer.org, accessed on 20 October 2023) was employed to search for *WRKY* genes in the *A. lancea* genome. Candidate WRKY protein sequences were submitted to the NCBI Conserved Domain Search (https://www.ncbi.nlm.nih.gov/Structure/cdd/wrpsb.cgi, accessed on 20 October 2023) for structural domain verification, and each sequence was manually inspected, resulting in the identification of 65 *WRKY* genes. Based on their chromosomal positions, these genes were designated “AlWRKYn,” where “Al” represents the Latin abbreviation for *A. lancea*, and “n” denotes their position on the chromosomes 1–12 from top to bottom. Physicochemical properties of the AlWRKY proteins, including isoelectric point, molecular weight, and instability index, were predicted using the ExPASy website.

### 2.3 Phylogenetic and sequence feature analysis

Multiple sequence alignments of AlWRKY protein sequences were conducted using MAFFT v.7 (Katoh and Standley, 2013), with manual refinement performed in BioEdit 7.0.9 (Hall, 1999). Then the AlWRKYs were then divided into different groups based on the classification of the *Arabidopsis thaliana* WRKY proteins sequences. Phylogenetic trees based on sequence alignment were constructed from the sequence alignments using IQ-TREE software with the maximum likelihood-based method, and the VT + R5 model was identified as the most appropriate (Nguyen et al., 2015). Finally, the phylogenetic tree was visualized and identified using the iTOL software (https://itol.embl.de/, accessed on 12 January 2024).

### 2.4 Conserved motifs and gene structure analysis

Conserved motifs of *A. lancea* WRKY protein sequences were identified using the MEME online tool (https://meme-suite.org/meme/tools/meme, accessed on 10 November 2023) (He et al., 2012), and the predicted results were visualized with TBtools. According to the gene annotation GFF files, the exon-intron structure was analyzed using the gene structure shower tool.

### 2.5 Analysis of cis-acting elements in promoters

The 2 kb upstream region sequences of *AlWRKY* genes were extracted and submitted to the PlantCARE website (http://bioinformatics.psb.ugent.be/webtools/plantcare/html/, accessed on 16 January 2024) for cis-element analysis. Cis-acting elements were subsequently visualized using TBtools (Lescot et al., 2002).

### 2.6 Chromosomal location, collinearity analysis, gene replication, and Ka/Ks analysis

Chromosomal positions of the *AlWRKY* genes were determined from the genomic structure annotation file and displayed using TBtools software (Wang et al., 2012). Subsequently, the Simple Ka/Ks Calculator tool was used to calculate the Ka/Ks ratios of the *AlWRKY* genes. Collinearity analysis was performed using two representative species—*Helianthus annuus* (a closely related Asteraceae member to *A. lancea*) to identify conserved syntenic blocks and *A. thaliana* (a distantly related eudicot model) to detect ancestral WRKY arrangements. Genomic data for *A. thaliana* and *Helianthus* were sourced from NCBI (Wang et al., 2010).

### 2.7 Gene co-expression and protein-protein interaction network analysis

Differentially expressed genes (DEGs) across various *A. lancea* tissues were identified using the DESeq2 v1.4.5 software, with a Q-value (Adjusted *P*-value) ≤ 0.05. Fragments per kilobase per million mapped reads (FPKM) of the genes were calculated using RSEM v1.3.1, and an expression atlas and gene co-expression network were generated using TBtools (Love et al., 2014). STRING (htKIN://string-db.org/) was used to construct the functional interaction network of the proteins.

### 2.8 Gene expression analysis using quantitative real-time PCR (qRT-qPCR)

Expression levels of terpene synthesis-related genes in *A. lancea* roots were assessed via qRT-PCR. The PCR primer sets are listed in Supplementary Table S1, with β-actin serving as the internal reference. Analysis was performed on an Agilent Mx3000P system (Agilent Technologies) using a 2x Realab Green PCR Fast Mixture Kit (LabLead Biotechnology, Beijing, China). Relative expression of the genes was calculated using the 2^−ΔΔCT^ method (Livak and Schmittgen, 2001).

### 2.9 Determination of volatile chemical composition using GC–MS

Dried rhizome samples of *A. lancea* were pulverized and sieved through a 50-mesh screen. Precisely, 0.1 g of powder was ultrasonically extracted with 3 mL of n-hexane for 30 min, cooled to room temperature, and replenished with fresh n-hexane. Following centrifugation, the supernatant was filtered through a 0.22 μm nylon membrane and analyzed via GC–MS using a DB-5 capillary column (30 m × 0.25 mm, 0.25 μm). GC–MS parameters aligned with those used in our previous study (Yu et al., 2019).

### 2.10 Molecular docking analysis of AlWRKY TFs with AlTPS promoters

Potential interactions between AlWRKY TFs (AlWRKY20 and AlWRKY37) and the promoter regions of *AlTPS* genes (*AlTPS70* and *AlTPS71*) were investigated using molecular docking analysis on the HDOCK server (Yan et al., 2017) with default parameters. Prior to docking simulations, the tertiary structures of AlWRKY20 and AlWRKY37 were predicted via homology modeling using the SWISS-MODEL web server (https://swissmodel.expasy.org/). Based on established criteria (Yan et al., 2017; Castillo-Zeledón et al., 2023), docking poses with scores <−200 and confidence scores >0.7 were considered to represent high-affinity binding interactions. Results were analyzed and visualized using PyMOL (version 3.1.3.1).

### 2.11 Cloning of AlWRKYs and subcellular localization assay

To analyze the subcellular localization of AlWRKY20 and AlWRKY37 proteins, we first predicted their localization using two widely used online tools, WoLF PSORT (https://www.genscript.com/wolf-psort.html) and CELLO (https://cello.life.nctu.edu.tw/). Subsequently, the open reading frames (ORFs) of these AlWRKYs were fused to the pBI121-GFP vector. Fusion plasmids and an empty vector were then transferred into GV3101 *Agrobacterium tumefaciens* in *Nicotiana benthamiana* leaves via *Agrobacterium*-mediated transformation. GFP signals were observed 2–3 days post-infection using a laser scanning microscope (LSM 900, ZEISS, Germany) and nuclei were stained with DAPI.

## 3 Results

### 3.1 Identification of the *WRKY* genes in *A. lancea*

A total of 65 *WRKY* genes were identified in *A. lancea* and designated *AlWRKY01* through *AlWRKY65* based on their chromosomal locations. Comprehensive data for these genes were presented, including gene ID, gene name, amino acid numbers, molecular weight (MW), isoelectric point (PI), and instability index, were compiled for these genes. The fundamental physical and chemical properties of the samples are listed in Table 1. The WRKY proteins ranged in length from 100 to 1,631 amino acids (AAs), with molecular weights spanning from 11284.44 to 183204.79 Da, and isoelectric points ranging from 5.00 to 10.02. Protein structure analyses confirmed that these selected proteins contained a complete WRKY domain or zinc finger structure.

**TABLE 1:** Information on the WRKY transcription factor family of *A. lancea*.

Gene ID	Gene number	Chromosome	AA	MW	PI	Instability index
AtL_chr01G0025.1	AlWRKY01	Chr01	532	57886.08	7.20	48.00
AtL_chr01G0160.1	AlWRKY02	Chr01	680	75186.51	5.59	55.50
AtL_chr01G0271.1	AlWRKY03	Chr01	235	25980.28	9.58	51.91
AtL_chr01G2058.1	AlWRKY04	Chr01	274	31256.00	9.06	56.74
AtL_chr01G2653.1	AlWRKY05	Chr01	181	20392.44	5.00	41.56
AtL_chr01G3249.1	AlWRKY06	Chr01	880	100549.70	5.65	45.05
AtL_chr01G3307.1	AlWRKY07	Chr01	507	56104.04	6.04	54.99
AtL_chr01G4528.1	AlWRKY08	Chr01	369	40018.30	9.35	63.46
AtL_chr01G4644.1	AlWRKY09	Chr01	339	38518.62	5.91	54.40
AtL_chr01G5494.1	AlWRKY10	Chr01	263	30235.66	5.14	44.87
AtL_chr01G5945.1	AlWRKY11	Chr01	215	24506.15	6.74	41.17
AtL_chr01G6720.1	AlWRKY12	Chr01	462	50491.93	6.89	47.53
AtL_chr02G2963.1	AlWRKY13	Chr02	319	34819.56	9.47	58.91
AtL_chr02G3000.1	AlWRKY14	Chr02	282	30663.47	9.53	54.05
AtL_chr02G3440.1	AlWRKY15	Chr02	388	41860.63	6.38	58.95
AtL_chr02G3890.1	AlWRKY16	Chr02	240	28342.32	8.71	51.39
AtL_chr02G4101.1	AlWRKY17	Chr02	341	36926.68	6.14	60.57
AtL_chr02G4949.1	AlWRKY18	Chr02	374	41109.43	5.69	51.72
AtL_chr03G1343.1	AlWRKY19	Chr03	399	43834.75	5.92	52.00
AtL_chr03G3612.1	AlWRKY20	Chr03	344	38049.30	7.63	46.72
AtL_chr03G5032.1	AlWRKY21	Chr03	418	45988.03	6.81	63.01
AtL_chr03G5397.1	AlWRKY22	Chr03	345	38348.40	9.73	50.33
AtL_chr03G5723.1	AlWRKY23	Chr03	261	30389.79	6.21	50.39
AtL_chr04G2427.1	AlWRKY24	Chr04	253	29047.83	9.60	41.93
AtL_chr04G3832.1	AlWRKY25	Chr04	1631	183204.80	9.13	39.82
AtL_chr04G4988.1	AlWRKY26	Chr04	333	37489.05	6.46	58.27
AtL_chr04G4990.1	AlWRKY27	Chr04	268	30727.79	9.25	43.86
AtL_chr04G4991.1	AlWRKY28	Chr04	265	30344.56	9.06	40.85
AtL_chr04G5001.1	AlWRKY29	Chr04	568	63434.97	7.60	55.83
AtL_chr04G5406.1	AlWRKY30	Chr04	299	33181.41	5.00	52.35
AtL_chr05G2214.1	AlWRKY31	Chr05	387	41673.59	9.81	52.77
AtL_chr05G2216.1	AlWRKY32	Chr05	553	60494.81	7.12	48.62
AtL_chr05G2939.1	AlWRKY33	Chr05	673	72933.85	6.07	55.09
AtL_chr05G3510.1	AlWRKY34	Chr05	340	36909.74	9.61	47.58
AtL_chr05G4150.1	AlWRKY35	Chr05	300	33603.72	8.75	46.72
AtL_chr06G1723.1	AlWRKY36	Chr06	450	49533.75	7.34	60.02
AtL_chr06G2402.1	AlWRKY37	Chr06	290	32150.66	5.50	58.24
AtL_chr06G4662.1	AlWRKY38	Chr06	454	51394.87	7.15	56.94
AtL_chr06G4798.1	AlWRKY39	Chr06	317	35840.96	6.37	57.70
AtL_chr07G0319.1	AlWRKY40	Chr07	562	61150.23	7.05	44.73
AtL_chr07G2792.1	AlWRKY41	Chr07	275	29625.43	9.94	59.50
AtL_chr07G3147.1	AlWRKY42	Chr07	476	52093.08	6.66	54.45
AtL_chr08G0648.1	AlWRKY43	Chr08	336	37667.46	5.42	51.99
AtL_chr08G0917.1	AlWRKY44	Chr08	111	12087.03	9.41	42.13
AtL_chr08G1295.1	AlWRKY45	Chr08	243	28018.56	6.08	47.36
AtL_chr08G1936.1	AlWRKY46	Chr08	249	28160.07	5.15	52.85
AtL_chr08G1983.1	AlWRKY47	Chr08	214	23926.45	5.59	60.15
AtL_chr08G4019.1	AlWRKY48	Chr08	300	32710.03	6.00	49.95
AtL_chr08G4050.1	AlWRKY49	Chr08	469	51054.88	6.84	64.04
AtL_chr09G2953.1	AlWRKY50	Chr09	281	31682.32	6.86	57.75
AtL_chr09G3489.1	AlWRKY51	Chr09	388	42086.36	5.62	49.31
AtL_chr09G4071.1	AlWRKY52	Chr09	319	36890.85	9.34	40.71
AtL_chr09G4073.1	AlWRKY53	Chr09	222	25317.04	5.60	42.93
AtL_chr09G4591.1	AlWRKY54	Chr09	281	30875.97	10.02	57.85
AtL_chr10G0527.1	AlWRKY55	Chr10	391	43459.67	9.16	50.71
AtL_chr10G0528.1	AlWRKY56	Chr10	100	11284.44	7.73	38.78
AtL_chr10G0824.1	AlWRKY57	Chr10	284	31555.80	5.27	67.40
AtL_chr10G1271.1	AlWRKY58	Chr10	167	18613.72	7.00	57.76
AtL_chr10G1720.1	AlWRKY59	Chr10	339	37287.11	6.11	52.80
AtL_chr10G2771.1	AlWRKY60	Chr10	304	33162.48	6.60	57.41
AtL_chr10G2786.1	AlWRKY61	Chr10	397	43255.14	8.88	46.52
AtL_chr11G1180.1	AlWRKY62	Chr11	344	39166.32	8.01	48.21
AtL_chr11G1667.1	AlWRKY63	Chr11	167	18527.59	7.00	59.42
AtL_chr11G3344.1	AlWRKY64	Chr11	373	41823.78	6.33	62.72
AtL_chr12G1801.1	AlWRKY65	Chr12	297	33369.06	5.67	55.99

### 3.2 Phylogenetic analysis and classification of *AlWRKYs*

**FIGURE 1:**
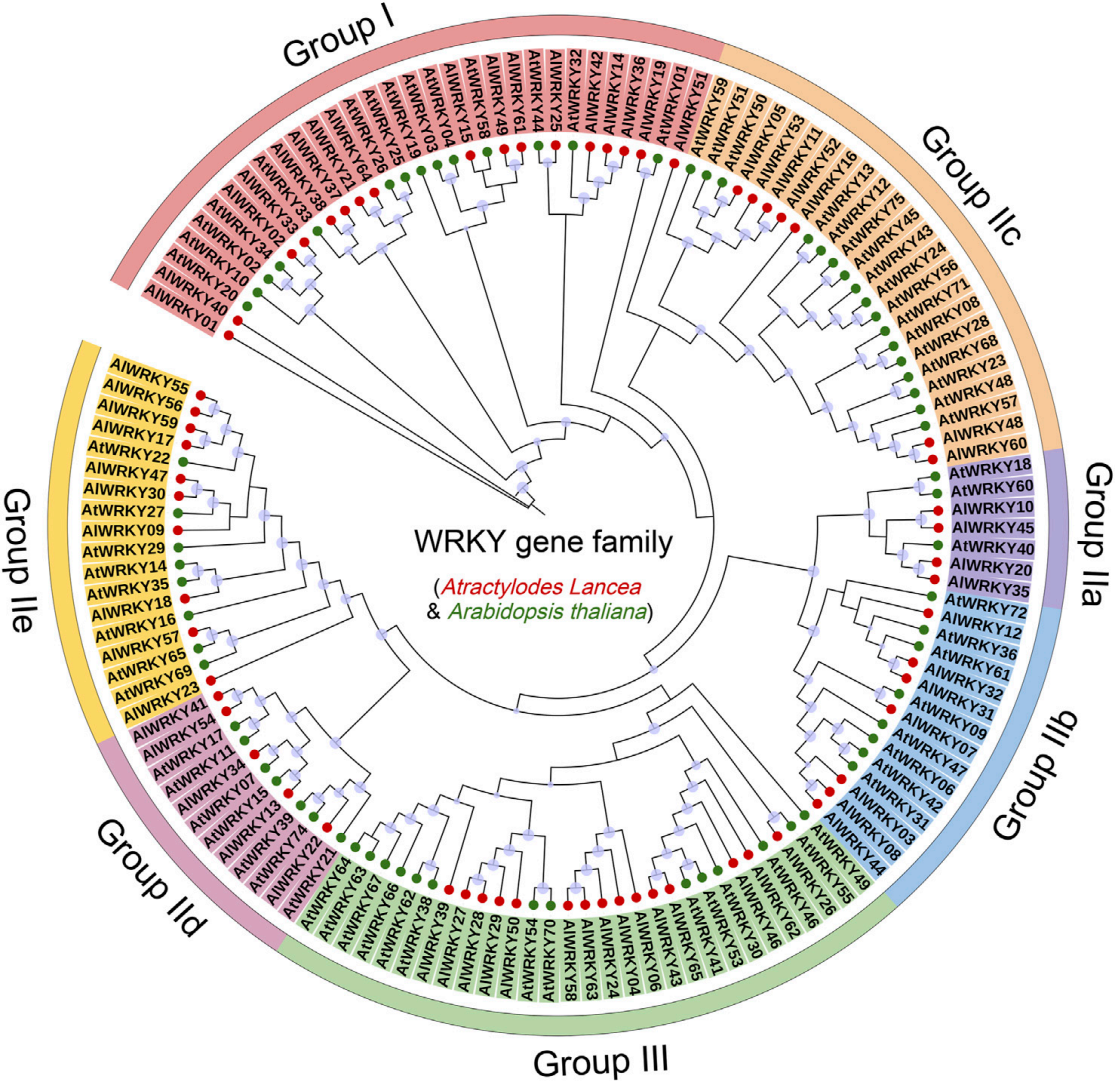
The maximum likelihood phylogenetic tree of WRKY protein of *Arabidopsis* and *A. lancea*. Red circles, *A. lancea* WRKY proteins; green circles, *Arabidopsis* WRKY proteins; light purple dot, a bootstrap value of ≥70, the larger the dot, the higher the value. The WRKY protein sequences are shown in Supplementary Table S2.

A circular phylogenetic tree comprising 65 *A. lancea WRKY* genes was constructed using the maximum-likelihood (ML) method to classify and elucidate the evolutionary relationships among the *AlWRKY* genes. Seventy-one *AtWRKY* genes from *A. thaliana,* representing distinct classification groups, served as references (Li et al., 2019). The AlWRKY proteins were categorized into three groups: Group I (17 members), Group II (33 members), and Group III (15 members) (Figure 1; Supplementary Table S2). Group II was further divided into five subgroups—IIa (four members), IIb (seven members), IIc (seven members), IId (five members), and IIe (ten members). Consequently, all 65 *AlWRKY* genes were systematically classified into three primary groups. Of the 17 AlWRKY proteins in Group I, only six possessed two WRKY domains. Phylogenetic analysis of full-length WRKY proteins revealed that 11 *AlWRKY* genes (*AlWRKY02, AlWRKY14, AlWRKY15, AlWRKY19, AlWRKY21, AlWRKY33, AlWRKY37, AlWRKY49, AlWRKY51, AlWRKY61, and AlWRKY64*) with incomplete or absent C-terminal WRKY structures were classified into Group I. In contrast, Group II included 33 WRKY proteins, each harboring either a single WRKY domain or a zinc finger structure. This group was further divided into five subgroups, distinguished by variations in their zinc finger structures. Subgroup IIa contains the CX5CPV(T/A)KKKVQ motif; subgroup IIb contains the CX5CPVRKQ(H)VQ; subgroup IIc has CX4C; subgroup IId features CX5CP(K)ARKH(R)VE(Q); and subgroup IIe includes CX5CXAR(K)K(R)VE. Group III comprised 15 proteins, each with a single WRKY domain and a C2HC zinc finger structure (C-X7-CX23–31-H-X1-C) (Cheng et al., 2023). Notably, there were a few exceptions: AlWRKY02, AlWRKY14, AlWRKY15, AlWRKY37, and AlWRKY51 proteins, although structurally aligned with Group II, clustered with Group I members in the ML analysis. We hypothesized that these WRKY proteins lost their C-terminal WRKY domain during evolution; alternatively, the C-terminal region may have been inaccurately annotated.

Through multiple sequence alignment of WRKY domains from *A. lancea*, the structure of the highly conserved WRKY domain heptapeptide was identified as WRKYGQK (Supplementary Figure S1). Variants including WRKYGKK, WKKYGEK, WEKYGQT, and WKKYGTK were observed in subgroups IIc, IId, and III (Song X. M. et al., 2023; Liu Q. G. et al., 2023).

### 3.3 *AlWRKY* gene chromosomal locations, duplications, and synteny analyses

**FIGURE 2:**
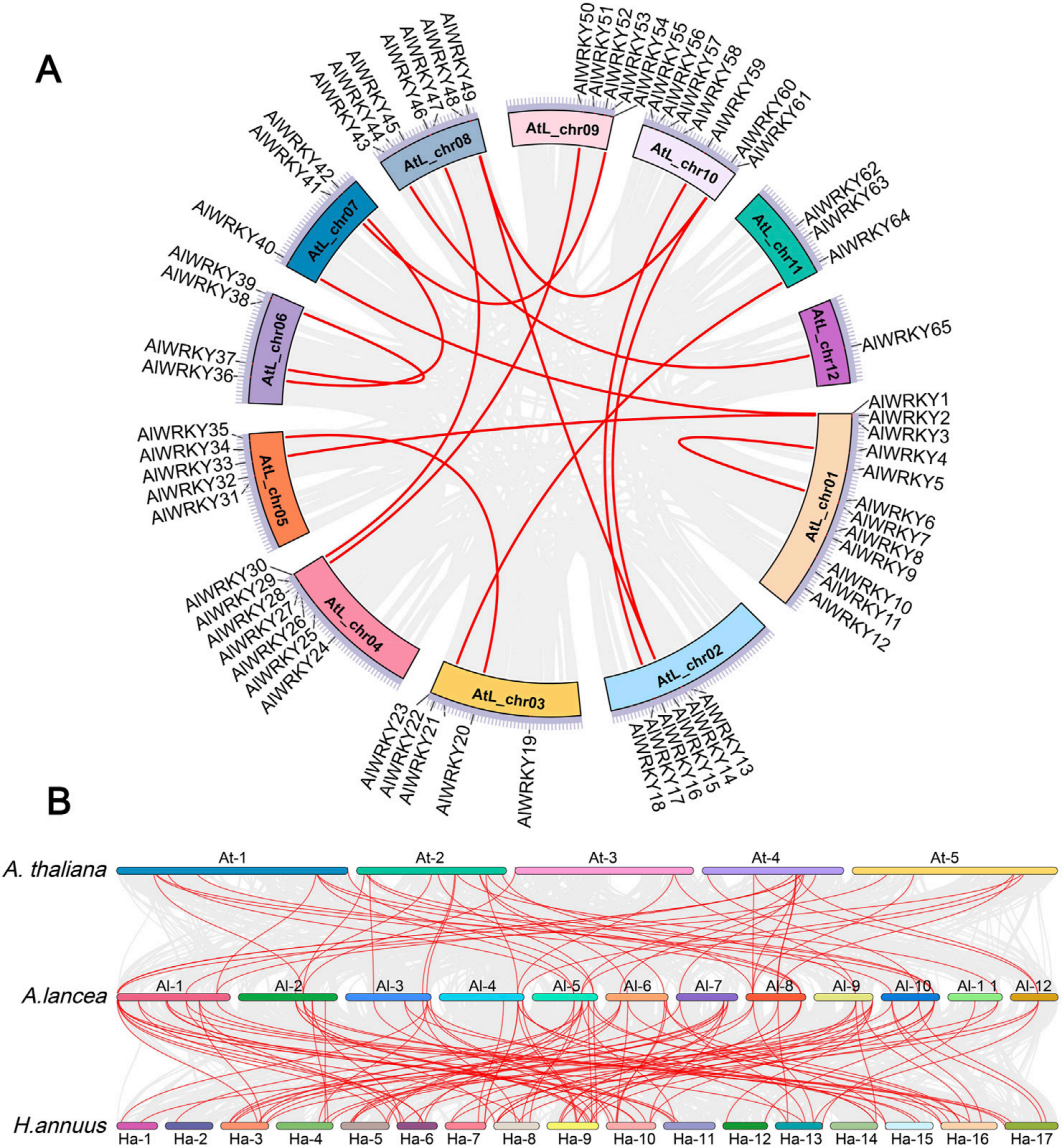
Collinearity of *AlWRKYs*. The collinearity relationships are marked with the red line. **(A)** The collinearity relationships of *AlWRKYs* within *A. lancea*. **(B)** The collinearity relationships of *WRKYs* between *A. lancea*, *A. thaliana*, and *H. annuus*.

The genomic distribution of *AlWRKY* genes was examined by mapping the approximate positions of the 65 AlWRKYs on the twelve chromosomes of *A. lancea*. As illustrated in Supplementary Figure S2, the distribution of *AlWRKY* genes across chromosomes was uneven. Specifically, chromosomes 3, 5, and 9 each harbored six genes, while chromosomes 4, 8, and 10 contained seven *AlWRKY* genes each. In contrast, chromosome 12 contained only a single gene, AlWRKY65. Segmental and tandem duplications are recognized as significant contributors to the expansion of plant gene families. To investigate the evolutionary regulation of the *A. lancea* WRKY gene family, segmental and tandem duplications of the 65 *AlWRKY* genes were analyzed using TBtools and MCScanX. The analysis identified four genes involved in tandem duplications: *AlWRKY27* and *AlWRKY28*, as well as *AlWRKY55* and *AlWRKY56* (Figure 2A). Furthermore, the parameters Ks (synonymous substitution rate) and Ka (nonsynonymous substitution rate) for duplication events were computed using the Simple Ka/Ks Calculator available in TBtools. The Ka/Ks ratio could not be determined for only one pair of *AlWRKY* segmental duplications (*AlWRKY15* and *AlWRKY60*), while the Ka/Ks ratios for the remaining 15 pairs of *AlWRKY* tandem duplications were found to be less than 1. This suggests that these *AlWRKY* gene pairs have undergone purifying selection.

To further explore the molecular evolutionary relationships between species, *H. annuus* and *A. thaliana* were used to perform an interspecies collinearity analysis of the *A. lancea* WRKY family (Figure 2B). The results showed that 46 and 107 pairs of *AlWRKY* genes exhibited collinear relationships with *WRKY* genes in *A. thaliana* and *H. annuus*, respectively. These results indicated that the *AlWRKY* genes exhibited higher homology with Asteraceae (*H. annuus*), potentially attributable to a close genetic relationship.

### 3.4 Gene structure and motif composition of *AlWRKYs*

**FIGURE 3:**
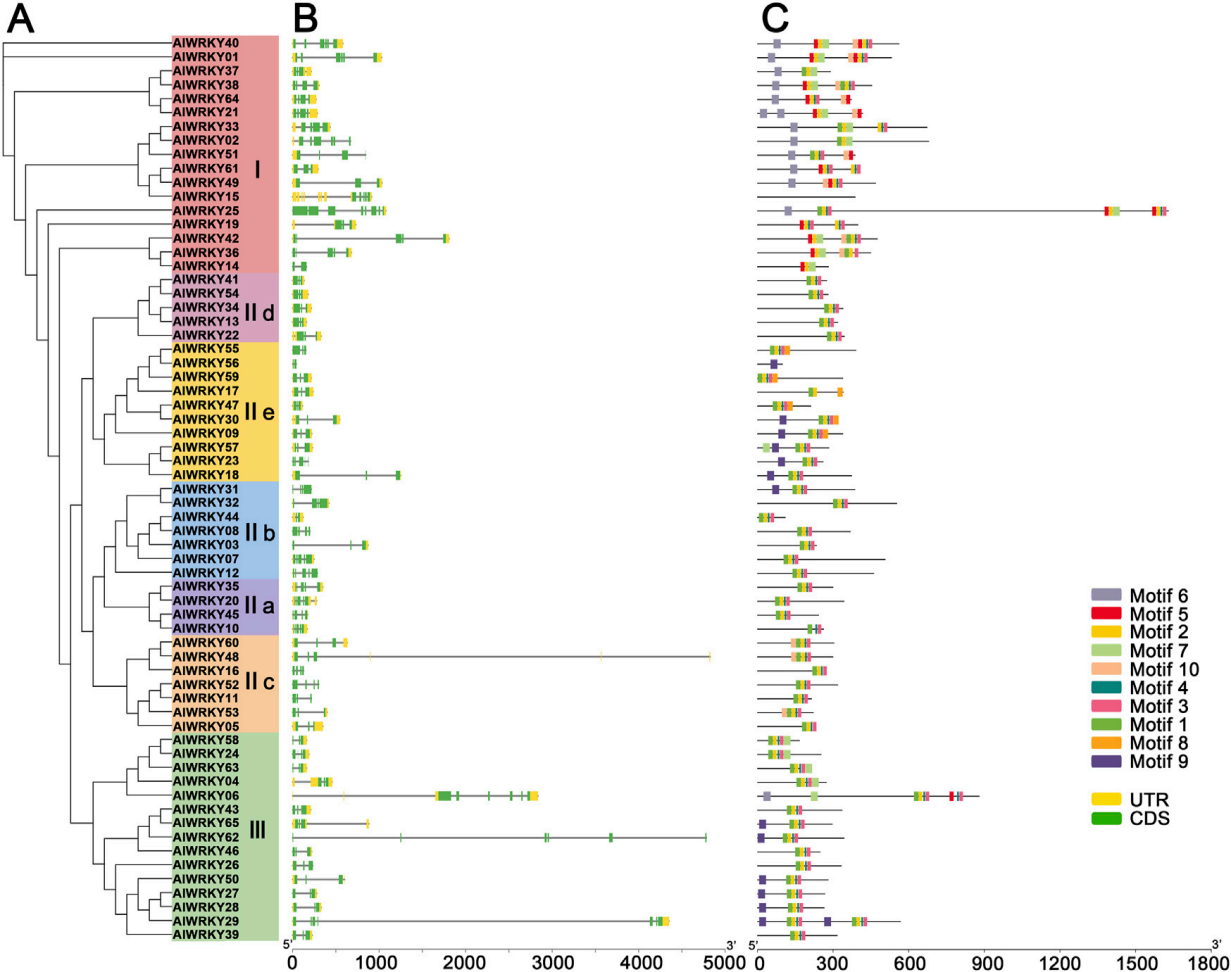
The grouping, gene structure and motif analyses of the AlWRKY family. **(A)** The maximum likelihood phylogenetic includes 65 AlWRKY proteins from *A. lancea*, which are clustered into 7 subgroups, sequentially designated as Ⅰ, Ⅱa, Ⅱb, Ⅱc, Ⅱd, Ⅱe and Ⅲ. **(B)** Exon-intron structure of *A. lancea WRKY* genes. Yellow boxes indicate untranslated 5′- and 3′-regions; green boxes indicate exons; black lines indicate introns. **(C)** The motif composition of *A. lancea* WRKY proteins. 10 different motifs are displayed in different colored boxes. The length of protein can be estimated using the scale at the bottom.

To gain deeper insights into the critical role of exon-intron structural features in the evolution of *A. lancea* gene families, the structure of *AlWRKY* genes was obtained through Visualize Gene Structure analysis (Figure 3B). All 65 *WRKY* genes were interrupted by introns, with the number of exons ranging from two to eight. Specifically, subgroups Ⅱa—IIe contained two to five exons, Group I had two to eight, and Group III had three to seven.

To further investigate the gene structure of *AlWRKY*, conserved motifs in AlWRKY proteins were predicted using MEME to assess functional regions (Figure 3C; Supplementary Table S3). Ten conserved motifs were identified and designated as motifs 1–10. Notably, motifs 1 and 5 included the heptapeptide sequence WRKYGQK, with most AlWRKY proteins possessing one or two WRKYGQK motifs. Genes within the same group or subgroup exhibited similar motif composition, suggesting functional conservation. For instance, motif 9 was predominantly found in subgroups IIe and III, whereas motif 10 was primarily observed in subgroups IIc and I. Additionally, motifs 5 and 6 were mainly distributed within group I.

### 3.5 Cis elements analysis of *AlWRKY* genes

**FIGURE 4:**
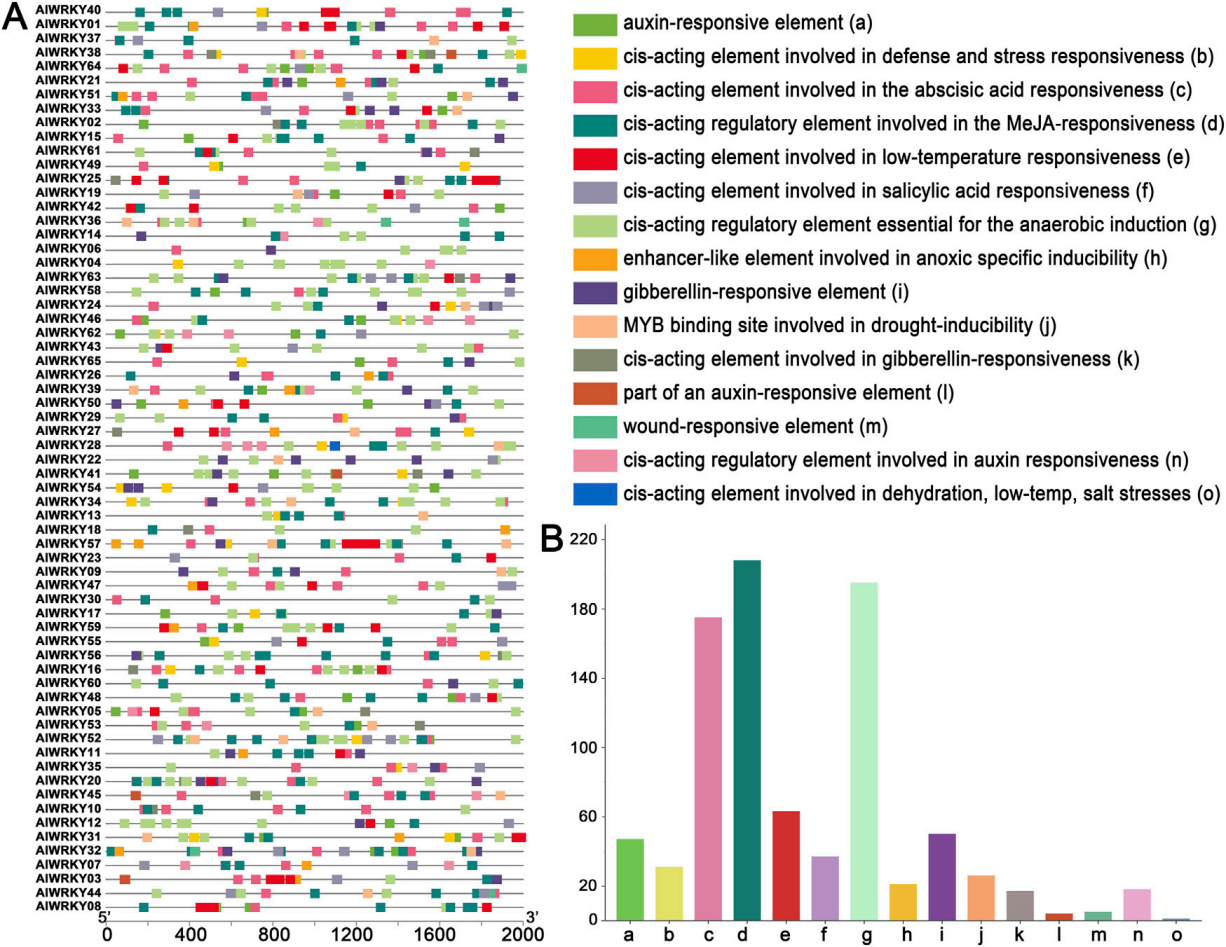
Cis-acting elements analysis of the promoters of *A. lancea WRKY* genes. **(A)** cis-acting element distribution. **(B)** Number of cis-acting element genes.

Most biological processes are predicted to involve various metabolic pathways and to respond to stressful conditions. To further explore the evolution and potential functions of *AlWRKY* genes under abiotic stress, an analysis of the upstream 2.0 kb promoter regions of *AlWRKY* genes was conducted using the PlantCARE database (Figure 4; Supplementary Table S4). The promoter regions of *AlWRKY* genes contain seven stress-responsive elements, including the TC-rich repeats (a cis-acting element involved in defense and stress responsiveness), the LTR (cis-acting element associated with low-temperature responsiveness), the ARE (cis-acting regulatory element essential for the anaerobic induction), the GC-motif (an enhancer-like element involved in anoxic specific inducibility), the DRE element (cis-acting element related to dehydration, low-temperature and salt stresses), the MBS element (a MYB binding site associated with drought inducibility), and the WUN-motif (a wound-responsive element), among others. ARE elements were the most abundant in the promoter regions of the *AlWRKY* genes, accounting for 57% of the total. LTR elements were identified in 30 promoters, whereas WUN-motif elements were found in four *AlWRKY* genes. Notably, AlWRKY18 contains a single DRE motif. Furthermore, five hormone-responsive cis-elements were identified: those involved in MeJA, abscisic acid, gibberellin, auxin, and salicylic acid responsiveness. Among these, the CGTCA-motif elements were present in 65 *AlWRKY* genes, representing 46% of the abiotic stress-related elements, followed by abscisic acid responsive elements (ABREs), which accounted for 27%.

### 3.6 Interaction analysis of specific AlWRKY proteins

To gain a deeper insight into the biological roles and the intricate regulatory networks associated with *AlWRKYs*, the potential protein-protein interactions (PPIs) among these proteins were forecasted employing an orthology-based approach. The outcomes revealed that 20 of the AlWRKY proteins shared orthologous connections with their counterparts in Arabidopsis (Supplementary Figure S3). We identified five interacting proteins with high-confidence interactions (score >0.7), including VQ proteins (such as Meckel syndrome, type 1 [MKS1] and sigma factor binding protein 1 [SIB1]), which are implicated in the regulation of plant defense responses, probable NADH dehydrogenase kinase F28J15.12 and T17B22.21, and TIFY6A proteins involved in repress transcription of jasmonate-responsive genes. The AlWRKY21 protein is highly homologous to *Arabidopsis* WRKY33, suggesting that it may have stronger interactions with most plant defense proteins (MKS1 and SIB1). Moreover, AlWRKY48 showed a close relationship with TIFY6A, a known repressor of jasmonate responses.

### 3.7 Expression patterns of *AlWRKY* genes in different organs of two chemotypes of *A. lancea*

**FIGURE 5:**
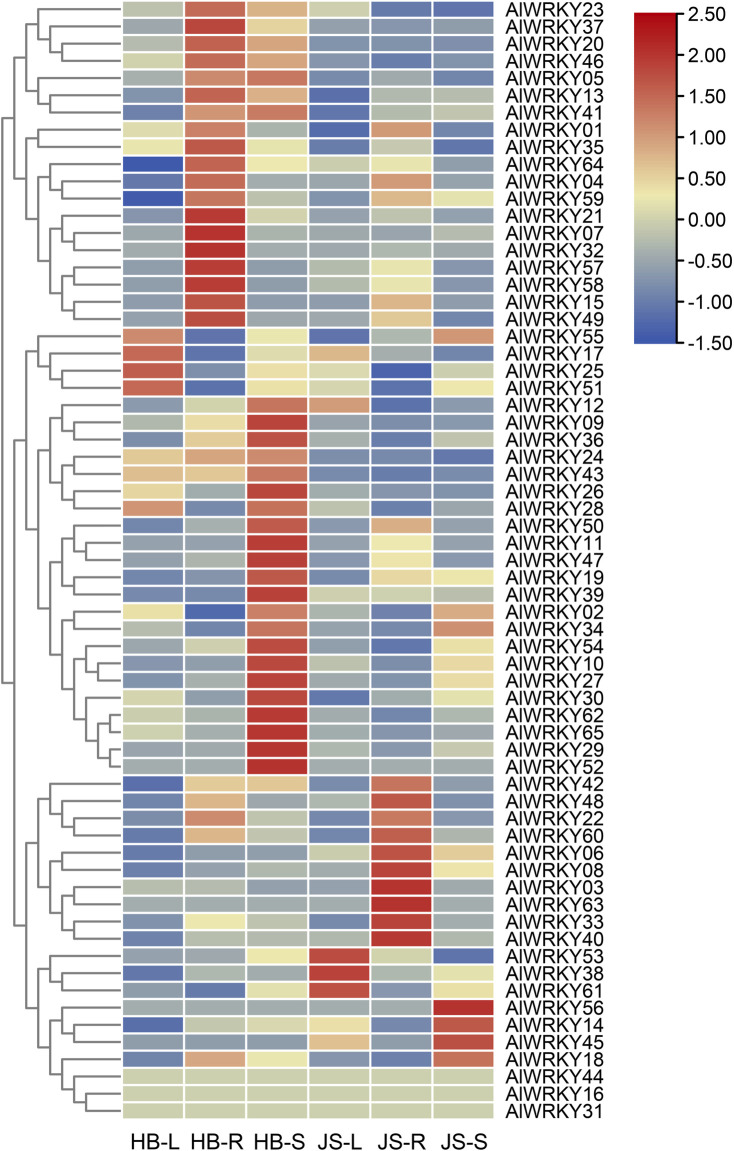
Heatmap of *AlWRKY* genes expression in leaves (L), rhizomes (R) and stem (S) of two chemotypes *A. lancea.* Hubei (HB), Jiangsu (JS).

WRKY TFs play a critical role in plant growth and development, often exhibiting tissue-specific expression patterns (Song H. et al., 2023). To identify WRKY TFs associated with biosynthesis of sesquiterpenoids, particularly hinesol and β-eudesmol, the expression profiles of the 65 *AlWRKY* genes were characterized in the rhizome, leaves, and stem tissues of *A. lancea* from the Hubei and Jiangsu regions (Figure 5; Supplementary Table S5). As sesquiterpenoids are predominantly found in rhizomes, and the concentrations of hinesol and β-eudesmol are significantly higher in Hubei than in Jiangsu (Xu et al., 2016), two chemical types of *A. lancea* rhizomes were used for comparative analysis. Differential gene expression analysis identified 11 DEGs: *AlWRKY06*, *AlWRKY10*, *AlWRK13*, *AlWRKY18*, *AlWRKY20*, *AlWRKY21*, *AlWRKY32*, *AlWRKY36*, *AlWRKY37*, *AlWRKY40*, and *AlWRKY49*. Among these, *AlWRKY13*, *AlWRKY20*, *AlWRKY21*, *AlWRKY37* and *AlWRKY49* were highly expressed in rhizomes of *A. lancea* from Hubei, consistent with the distribution of sesquiterpenes. Therefore, these TFs may activate genes involved in terpenoid biosynthesis, thereby regulating the synthesis of related terpenoid metabolites in *A. lancea*.

### 3.8 Co-expression analysis of candidate *AlWRKY* and *AlTPS* genes

**FIGURE 6:**
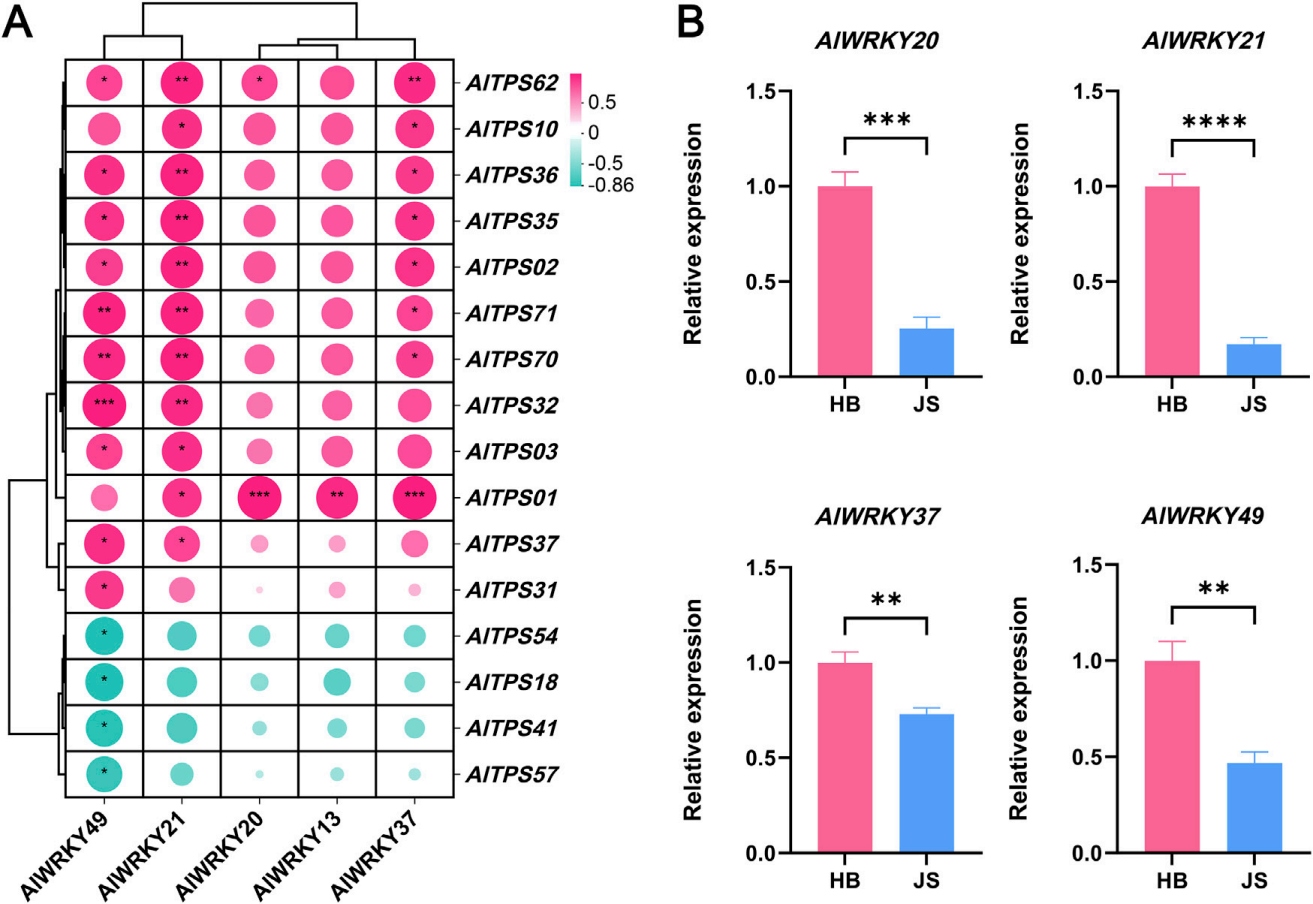
Correlation analysis of *AlWRKYs* and *AlTPSs* and expression profiling of candidate AlWRKYs in *A. lancea*. **(A)** *AlWRKYs* of *A. lancea* correlation with candidated *AlTPS* genes. **(B)** RT-qPCR validation of *AlWRKY* gene expression in Hubei (HB) and Jiangsu (JS). Statistical significance was assessed by Student’s t-test (*p < 0.05, **p < 0.01, ***p < 0.001).

Co-expression network analysis was conducted to identify genes exhibiting coordinated expression patterns across various samples. A co-expression network was constructed using *AlWRKY* and *AlTPS* genes from *A. lancea*. The *AlWRKY* unigene set was combined with the expression of candidate *AlTPS* genes to assume that WRKY unigenes may be involved in terpenoid biosynthesis. The transcription levels of the two co-expressed gene sets displayed similar expression profiles throughout the samples. Pearson’s correlation analysis was conducted between the five differentially expressed *AlWRKY* genes and all 74 *AlTPS* genes, revealing 16 *AlTPS* genes that showed significant correlations (|r| > 0.8, *p* < 0.05) with these AlWRKY TFs. Subsequently, the 16 *AlTPS* genes exhibiting positive correlations with differentially expressed *AlWRKY* genes were selected for co-expression network analysis, as illustrated in Figure 6A. The gene family members *AlWRKY21* and *AlWRKY49* in *A. lancea* were highly correlated with *AlTPS2*, *AlTPS32*, *AlTPS70* and *AlTPS71* expression, whereas *AlWRKY20*, *AlWRKY13* and *AlWRKY37* were positively correlated with *AlTPS1* expression. *AlWRKY49* was negatively correlated with *AlTPS18*, *AlTPS41*, *AlTPS54*, and *AlTPS57.*

### 3.9 Comparative analysis of volatile components and *AlWRKY* gene expression between two chemotypes of *A*. *lancea*

GC–MS analysis of two *A. lancea* chemotypes identified 18 major volatile compounds, predominantly sesquiterpenoids, by comparison with the NIST mass spectral library. As summarized in Table 2, both chemotypes shared several common compounds including berkheyaradulene, β-caryophyllene, γ-elemene, humulene, β-sesquiphellandrene, cubenol, γ-eudesmol, hinesol, β-eudesmol, and atractylodin. However, distinct chemotypic differences were observed: β-elemene, β-himachalene, β-selinene, selina-3,7(11)-diene, and atractylone were absent in Yingshan populations, while zingiberene, elemol, and aristolene were undetectable in Nanjing specimens. Notably, hinesol and β-eudesmol collectively constituted more than 60% of volatile oils in Yingshan chemotypes, compared to only 3%–4% in Nanjing samples. Conversely, atractylon and atractylodin dominated the Nanjing chemotypes (exceeding 50% of total volatiles) but were nearly negligible (0%–1%) in the Yingshan populations.

**TABLE 2:** Contents of the main components obtained from the rhizome of *A. lancea*.

No.	Retention time (min)	Molecular formula	Molecular weight	CAS	Compounds	Relative content (Mean ± SD, %)	
						HB	JS
1	29.06	C_15_H_24_	204	515-13-9	β-Elemene	0.00b	0.15 ± 0.02a
2	29.23	C_15_H_24_	204	65372-78-3	Berkheyaradulene	0.04 ± 0.07b	5.22 ± 0.47a
3	30.55	C_15_H_24_	204	87-44-5	β-Caryophyllene	0.04 ± 0.01b	4.49 ± 0.57a
4	30.76	C_15_H_24_	204	29873-99-2	γ-Elemene	0.01 ± 0.02b	3.33 ± 0.34a
5	32.07	C_15_H_24_	204	6753-98-6	Humulene	0.01 ± 0.003b	1.30 ± 0.12a
6	32.75	C_15_H_24_	204	1461-03-6	β-Himachalene	0.00b	0.39 ± 0.05a
7	33.44	C_15_H_24_	204	495-60-3	Zingiberene	0.04 ± 0.04	0.00
8	33.48	C_15_H_24_	204	17066-67-0	β-Selinene	0.00b	1.35 ± 0.07a
9	34.66	C_15_H_24_	204	20307-83-9	β-Sesquiphellandrene	0.02 ± 0.01b	0.11 ± 0.01a
10	35.40	C_15_H_24_	204	6813-21-4	Selina-3,7 (11)-diene	0.00b	3.08 ± 0.19a
11	35.74	C_15_H_26_O	222	639-99-6	Elemol	2.23 ± 0.42a	0.00b
12	38.49	C_15_H_26_O	222	21284-22-0	Cubenol	0.34 ± 0.12a	0.01 ± 0.02b
13	39.15	C_15_H_26_O	222	1209-71-8	γ-Eudesmol	6.98 ± 1.68a	0.01 ± 0.01b
14	39.29	C_15_H_24_	204	6831-16-9	Aristolene	0.97 ± 0.24a	0.00b
15	39.58	C_15_H_26_O	222	23811-08-7	Hinesol	55.69 ± 7.61a	0.42 ± 0.13b
16	40.20	C_15_H_26_O	222	473-15-4	β-Eudesmol	5.43 ± 3.66	3.51 ± 0.32
17	40.42	C_15_H_20_O	216	6989-21-5	Atractylon	0.00b	35.66 ± 0.54a
18	45.81	C_13_H_10_O	182	55290-63-6	Atractylodin	0.01 ± 0.01	16.80 ± 2.03a

Lowercase letters denote statistically significant differences (Student’s t-test, *p* < 0.05).

Quantitative reverse transcription polymerase chain reaction (RT-PCR) was performed to verify the hub *WRKY* genes in *A. lancea*: *AlWRKY13*, *AlWRKY20*, *AlWRKY21*, *AlWRKY37*, and *AlWRKY49*. Rhizomes from two *A. lancea* chemotypes, sourced from Hubei and Jiangsu, were used for the qPCR validation. As shown in Figure 6B, expression levels of *AlWRKY20*, *AlWRKY21*, *AlWRKY37* and *AlWRKY49* genes were higher in Hubei rhizomes than in Jiangsu rhizomes, consistent with the distribution of sesquiterpenes (hinesol, γ-eudesmol, and elemol). No differential expression of *AlWRKY13* was observed between the two regions (Supplementary Figure S4).

### 3.10 Comparative analysis of volatile components and *AlWRKY*/*AlTPS* gene expression between MeJA-treated samples

**FIGURE 7:**
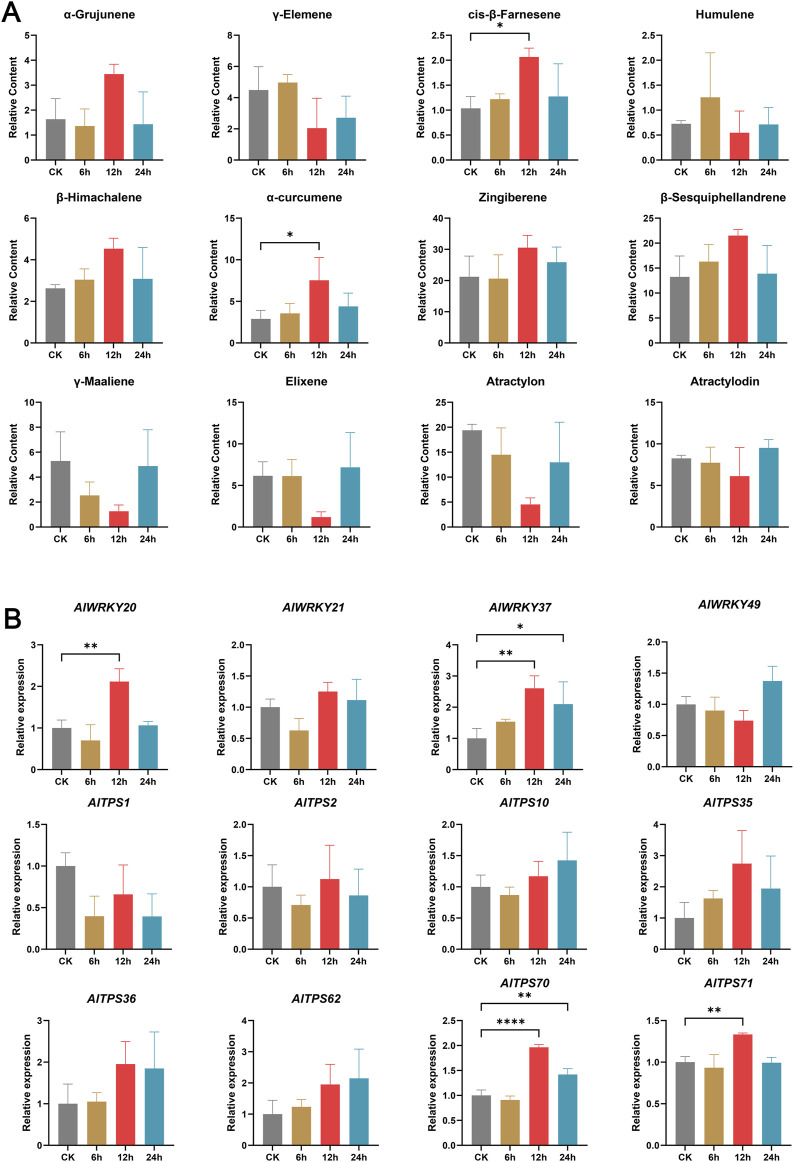
Dynamic changes of major components and expression patterns of AlWRKYs and AlTPSs in A. lancea rhizomes under MeJA treatment. **(A)** Dynamic changes of major components. **(B)** Expression patterns of AlWRKYs and AlTPSs. Columns and bars separately represent the means and standard deviation (n = 3), and the data was determined by One-way ANOVA (*p < 0.05, **p < 0.01, ****p < 0.0001).

To further validate the functions of these four genes, methyl jasmonate (MeJA) treatment was applied to *A. lancea* plants. The correlation between their differential expression patterns and corresponding chemical composition changes was analyzed to predict their putative biological roles. GC–MS analysis of *A. lancea* chemotypes treated with MeJA at different time points (0 h, 6 h, 12 h, and 24 h) identified 12 major volatile compounds, predominantly sesquiterpenoids (Figure 7A), by comparison with the NIST mass spectral database. The relative content of α-grujunene and zingiberene showed a “decrease-increase-decrease” trend, whereas cis-β-farnesene, β-himachalene, α-curcumene, and β-sesquiphellandrene exhibited an “initial increase followed by a decrease” pattern. In contrast, γ-maaliene, elixene, atractylon, and atractylodin displayed an “initial decrease followed by an increase” trend. In addition, γ-elemene and humulene demonstrated a “fluctuating (increase-decrease-increase)” pattern. Significant increases in cis-β-farnesene and α-curcumene were observed at 12 h compared with the control (CK) (*P* < 0.05).

RT-PCR analysis of MeJA-treated *A. lancea* samples revealed that expression levels of *AlWRKY21* and *AlWRKY49* remained unchanged across treatment durations, whereas *AlWRKY20* exhibited significantly higher expression at 12 h, and *AlWRKY37* showed elevated expression at both 12 h and 24 h compared with the CK (Figure 9B) (*p* < 0.05). Subsequently, eight TPS genes (*AlTPS1, AlTPS2, AlTPS10, AlTPS35, AlTPS36, AlTPS62, AlTPS70*, and *AlTPS71*) predicted to interact with *AlWRKY20* and *AlWRKY37* (Figure 6A), were examined. The results showed that the expression level of *AlTPS1* at 12 h was lower than that of the CK, whereas *AlTPS2*, *AlTPS10*, *AlTPS35*, *AlTPS36*, and *AlTPS62* exhibited higher expression levels than the CK at 12 h. In addition, *AlTPS70* showed significantly higher expression levels than CK at both 12 and 24 h, and *AlTPS71* was significantly upregulated compared to CK at 12 h (*p* < 0.05) (Figure 7B). Notably, the coordinated upregulation of *AlWRKY20, AlWRKY37, AlTPS70*, and *AlTPS71* at 12 h positively correlated with cis-β-farnesene and α-curcumene accumulation, suggesting that *AlWRKY20* and *AlWRKY37* likely promote the biosynthesis of cis-β-farnesene and α-curcumene through their regulatory effects on *AlTPS* gene expression.

### 3.11 Molecular docking analysis of AlWRKY TFs with AlTPS promoters

**FIGURE 8:**
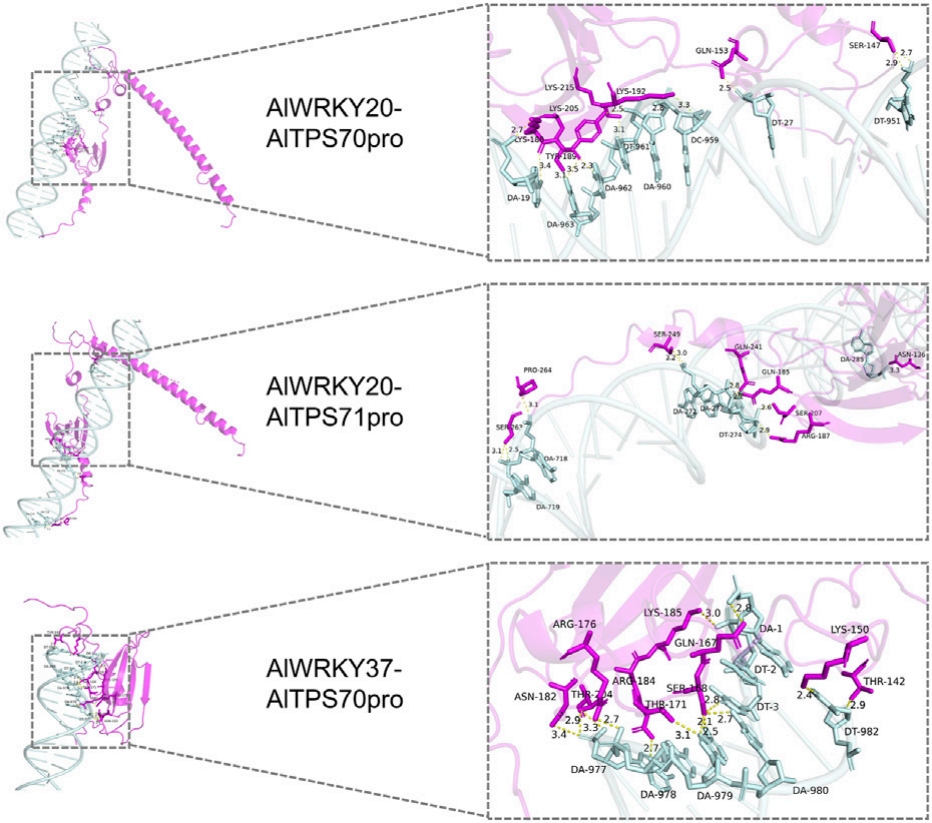
Molecular docking of AlWRKY transcription factors with AlTPS promoters. The interacting amino acid residues forming hydrogen bonds are depicted in magenta, nucleotide bases are shown in pale cyan, and hydrogen bond interactions are highlighted in yellow.

Homology modeling was performed for AlWRKY20 using A0A2J6JT24.1. A as the template, yielding good model quality with 87.04% sequence identity and a GMQE score of 0.63. Similarly, AlWRKY37 was modeled using A0A118K6T8.1. A as the template, achieving 83.97% sequence identity ansd a GMQE score of 0.54. Molecular docking analysis conducted with HDOCK revealed the following: (1) The AlWRKY37-AlTPS71 promoter complex exhibited poor binding potential with a docking score greater than −200 and confidence score less than 0.7; (2) in contrast, AlWRKY20 exhibited strong binding to both the AlTPS70 and AlTPS71 promoters, while AlWRKY37 showed good binding to the AlTPS70 promoter, all with docking scores less than −200 and confidence scores exceeding 0.7, indicating high reliability of these complex models (Supplementary Table S6). Structural analysis (Figure 8) demonstrated that: (1) Both the AlWRKY20-AlTPS70 and AlWRKY20-AlTPS71 promoter complexes formed 11 hydrogen bonds, respectively, with binding interfaces predominantly involving Lys, Gln, and Ser residues; (2) the AlWRKY37-AlTPS70 promoter interaction formed 15 hydrogen bonds, with the binding interface primarily composed of Thr, Lys, and Arg residues.

### 3.12 Cloning, bioinformatics and subcellular localization analysis of *AlWRKY20* and *AlWRKY37*

**FIGURE 9:**
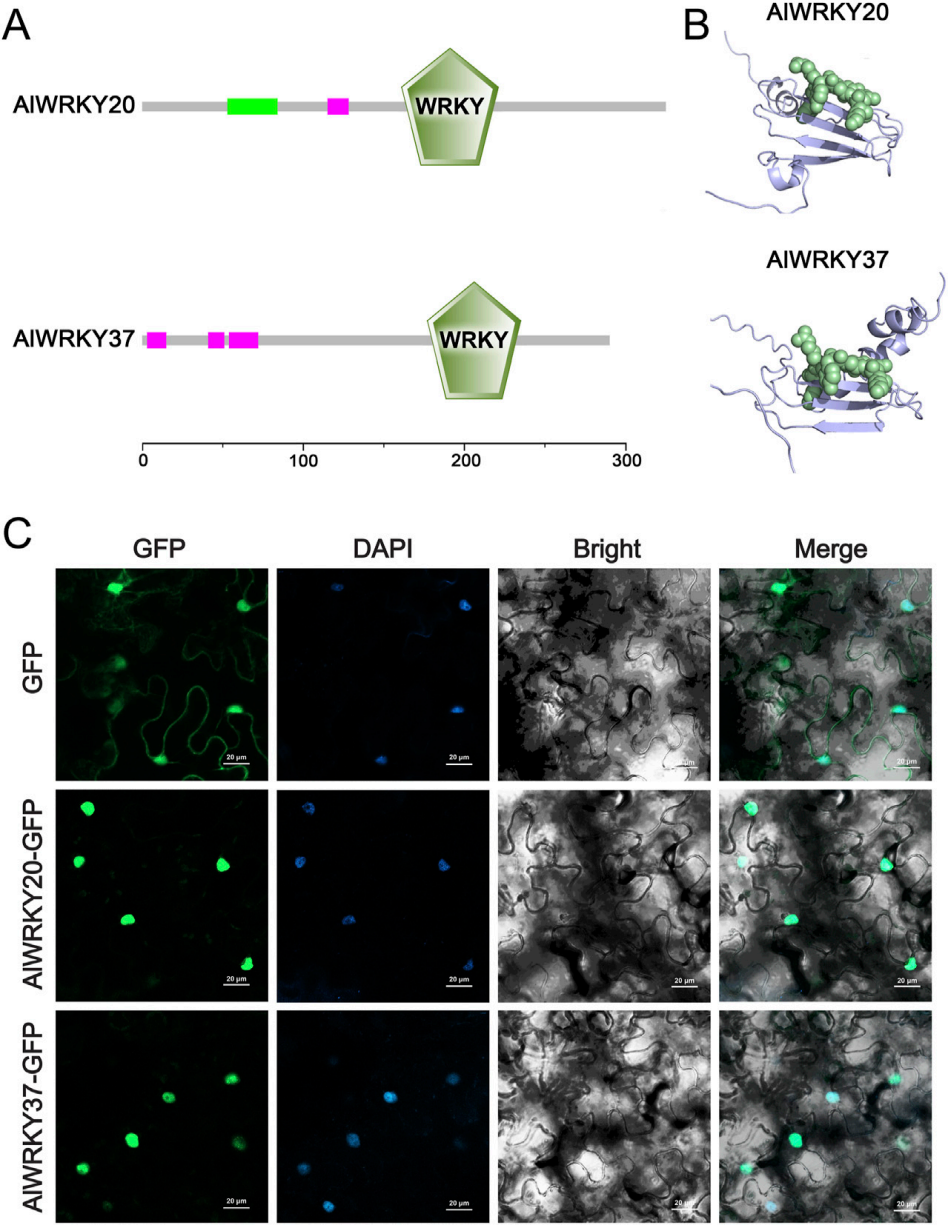
Conserved motif, protein tertiary structure analyses and subcellular localisation of *AlWRKY20* and *AlWRKY37* genes. **(A)** Conserved motif of these two genes. **(B)** Protein tertiary structure of these two genes. **(C)** Subcellular localisation of these two genes. GFP was used as the negative control. The green fluorescence indicates the location of fusion proteins. Scale bars = 20 µm.

Two candidate genes (*AlWRKY20* and *AlWRKY37*) were successfully cloned for subsequent functional studies. The ORFs of *AlWRKY20* and *AlWRKY37* were 1,035 bp and 873 bp, encoding proteins of 344 and 290 AAs, respectively (Figure 9A). Each of AlWRKY20 and AlWRKY37 possesses one WRKYGQK motif, the signature sequence of WRKY transcription factors (Figure 9A). The protein tertiary structures were constructed using the structures of *A. thaliana* WRKY proteins as models (Figure 9B).

Subcellular localization prediction analyses using WoLF PSORT and CELLO strongly suggested nuclear localization of AlWRKY20 and AlWRKY37 (Supplementary Table S7). To further study the subcellular localization of these two AlWRKY proteins, recombinant plasmids were constructed and transiently expressed in tobacco leaves with the empty GFP vector as a control. The results showed that the fluorescent signals of AlWRKY20-GFP and AlWRKY37-GFP fusion proteins were predominantly localized in the nucleus, consistent with previous predictions. The signal of 35S-GFP was detected in the nucleus and cytoplasm. Notably, the green fluorescence emitted from AlWRKY20-GFP and AlWRKY37-GFP fusion protein matched the blue fluorescence produced by DAPI staining of nuclei (Figure 9C), suggesting that AlWRKY20 and AlWRKY37 is a nucleus-localized protein.

## 4 Discussion

Recent years have seen a deepening understanding of the pharmacological effects of the major active components of *A. lancea* (Sun et al., 2022; Na-Bangchang et al., 2017), establishing it as one of the best-selling traditional Chinese medicines. WRKY TFs rank among the largest TF families and serve as key regulators of numerous plant processes. This family has been characterized across a wide range of model plants and medicinal plant species, including 71 *AtWRKY* genes in *A. thaliana* (Abdullah-Zawawi et al., 2021), 122 *AaWRKY* genes in *Artemisia annua* (Paolis et al., 2020), 63 *DoWRKY* genes in *Dendrobium officinale* (Wang et al., 2018), 64 *CeqWRKY* genes in *Casuarina equisetifolia* (Zhao et al., 2024) and 79 *WfWRKY* genes in weeping forsythia (Yang et al., 2023). In the present study, 65 *AlWRKY* genes were identified and the first genome-wide analysis of the WRKY gene family in *A. lancea* was performed. The WRKY gene family in *A. lancea* is relatively small compared with that of other medicinal plants. This contraction parallels observations in *Salvia miltiorrhiza* (Li et al., 2015), where metabolic specialization was associated with a reduction in regulatory genes.

Members of the WRKY protein family are defined by a conserved structural feature comprising the WRKYGQK motif and a zinc finger structure. Based on these attributes, the 65 AlWRKY proteins can be classified into three primary groups (I–III) and five subgroups (IIa, IIb, IIc, IId, and IIe). Group II contains the largest proportion of AlWRKY proteins. This classification is consistent with the findings for *Prunus sibirica* and *Neolamarckia cadamba* (Yu et al., 2024; Xu ZW. et al., 2023). Analysis of the core domain of AlWRKY proteins and the structure of *AlWRKY* genes revealed a strong correlation between motif structure and phylogenetic relationships, further supporting the classification observed in the *HuWRKY* gene family (Chen et al., 2022). In parallel, multiple sequence alignments of the conserved domains of 65 AlWRKY proteins identified four variants of the AlWRKY domain: WRKYGKK, WKKYGEK, WEKYGQT, and WRKFGQK. Notably, the WRKY domains of four AlWRKY proteins (AlWRKY05, AlWRKY11, AlWRKY52, and AlWRKY53) in Group IIc contained the heptapeptide variant WRKYGKK, a variation consistent with forms commonly observed in *Caragana korshinskii* and *Asteranae* (Liu J. H. et al., 2023; Guo et al., 2019). This suggests potential variability in the DNA-binding affinity associated with these variants (Chen et al., 2018). Variations in exon number, gene structure, and coding sequence (CDS) length among *AlWRKY* genes across different classifications, combined with the uneven distribution of gene numbers among various groups and subgroups and their irregular chromosomal localization, indicate that distinct *WRKY* genes may have undergone diverse evolutionary processes (Qiao et al., 2018).

In plant genomes, differentiation of *WRKY* genes has resulted in disparities in genes numbers within groups, with gene family expansion driven significantly by copy number expansion and tandem or local duplications (Cannon et al., 2004). Collinearity analysis identified two pairs of tandem duplications (*AlWRKY27* and *AlWRKY28*, *AlWRKY55*, and *AlWRKY56*) and 16 pairs of segmental duplications in the *A. lancea* WRKY gene family, a phenomenon likely contributing substantially to the expansion of this gene family, which is consistent with the situation in *Platycodon grandiflorus*, Zea mays, and *Cucumis sativus* (Yu et al., 2024; Hu et al., 2021; Chen et al., 2020)*.* Comparative transcriptomics revealed divergent expression patterns between tandem duplicates and non-duplicated WRKYs. *AlWRKY27* displays stem-specific expression, whereas *AlWRKY28* exhibits preferential expression in both leaves and stems, suggesting subfunctionalization between these paralogs. Phylogenetic similarities were observed between *AlWRKY27* and *AlWRKY28* as well as between *AlWRKY55* and *AlWRKY56*. Subsequent examination of cis-acting elements in their promoters revealed involvement in defense (TC-rich repeats), low-temperature responsiveness (LTR), and anaerobic induction (ARE). Collinearity analysis with other plants. demonstrated the existence of conserved *WRKY* genes in *A. lancea* that are evolutionarily related to those in other plants, such as *A. thaliana*, known as orthologous genes. Therefore, the functional analysis and validation of AlWRKYs can be guided by the functions of *WRKYs* in other plants.

WRKY proteins act as critical regulators of secondary metabolite production in various biological processes (Eulgem et al., 1999; Ulker and Somssich, 2004). Evidence suggests that specific WRKY proteins, either independently or in synergy with other TFs, play pivotal roles in the biosynthesis of valuable natural products (Hsin et al., 2022). Terpene synthase is a fundamental enzyme in terpene biosynthesis, with transcriptional levels of *TPS* genes involved in terpenoid biosynthesis modulated by WRKY TFs (Wei et al., 2023). Research on *A. annua* has indicated that *AaWRKY1* activates the expression of *AaADS* and *AaCYP71AV1* to control the production of artemisinin (Ma et al., 2009). In *L. cubeba*, *LcWRKY17* transactivates the promoters of the monoterpene synthase genes *LcTPS42*, contributing to monoterpene synthesis (Gao et al., 2023). A strong correlation has been observed between six *AvWRKY* unigenes and eight deduced *AvTPS* unigenes in *Amomum villosum*, indicating that these *WRKY* genes may play crucial roles in regulating terpene biosynthesis (He et al., 2018). In this study, MeJA induction (12 h) triggered coordinated expression of *AlWRKY20*/*AlWRKY37* and *AlTPS70*/*AlTPS71*, correlating with elevated cis-β-farnesene and α-curcumene accumulation alongside. Molecular docking confirmed binding of both AlWRKY20 and AlWRKY37 to *AlTPS* promoters. *TcWRKY47* from *Taxus chinensis* significantly upregulates taxol-biosynthesis-related genes (Zhang et al., 2018), and both *AlWRKY20* and *TcWRKY47* belong to Group IIa. Similarly, *SmWRKY2* in *S. miltiorrhiza* primarily enhances tanshinone biosynthesis by activating *SmCPS* (Deng et al., 2019), while *AlWRKY37* and *SmWRKY2* are classified under Group I. These findings indicated that *AlWRKY20* and *AlWRKY37* may be involved in the generation of sesquiterpenes through *AlTPS* gene modulation. However, further validation of the specific functions of *AlWRKY20* and *AlWRKY37* in terpenoid metabolism in *A. lancea* is needed to comprehensively understand their mechanisms of action.

## 5 Conclusion

This study provides the first comprehensive genome-wide analysis of WRKY transcription factors in Atractylodes lancea, identifying 65 *AlWRKY* genes with conserved domains. Phylogenetic classification revealed three major groups: Group I (17 members), Group II (33 members), and Group III (15 members). Tissue-specific expression profiling identified five rhizome-enriched *AlWRKY* genes that showed chemotype-dependent expression patterns in Hubei and Jiangsu populations. Multiple lines of evidence supporting that *AlWRKY20* and *AlWRKY37* play the potential regulatory roles in sesquiterpene biosynthesis regulation, as evidenced by their nuclear localization, co-expression with terpene synthase genes (*AlTPSs*), molecular docking, and response to MeJA treatment. These results suggest that *AlWRKY20* and *AlWRKY37* likely function as regulators of sesquiterpene biosynthesis, positively regulating cis-β-farnesene and α-curcumene production through *AlTPS* gene modulation. These findings not only contribute to elucidating the molecular mechanisms underlying WRKY-mediated regulation of terpenoid biosynthesis in *A. lancea* but also provide valuable genetic resources for future metabolic engineering efforts aimed at improving medicinal compound production in this important traditional herb. Further studies should focus on validating these regulatory networks through genetic transformation and detailed functional analyses.

## Data Availability

The original contributions presented in the study are included in the article/Supplementary Material, further inquiries can be directed to the corresponding author.
