# Supplementary material for: Genome-wide identification and expression analysis of the WRKY transcription factors related to sesquiterpenes biosynthesis in Atractylodes lancea
Source: Front Genet. 2025 May 15;16:1551991. doi: 10.3389/fgene.2025.1551991 (PMC12119531; doi:10.3389/fgene.2025.1551991)
Supplement: Supplementary file 2 [file Supplementaryfile1.doc]

**
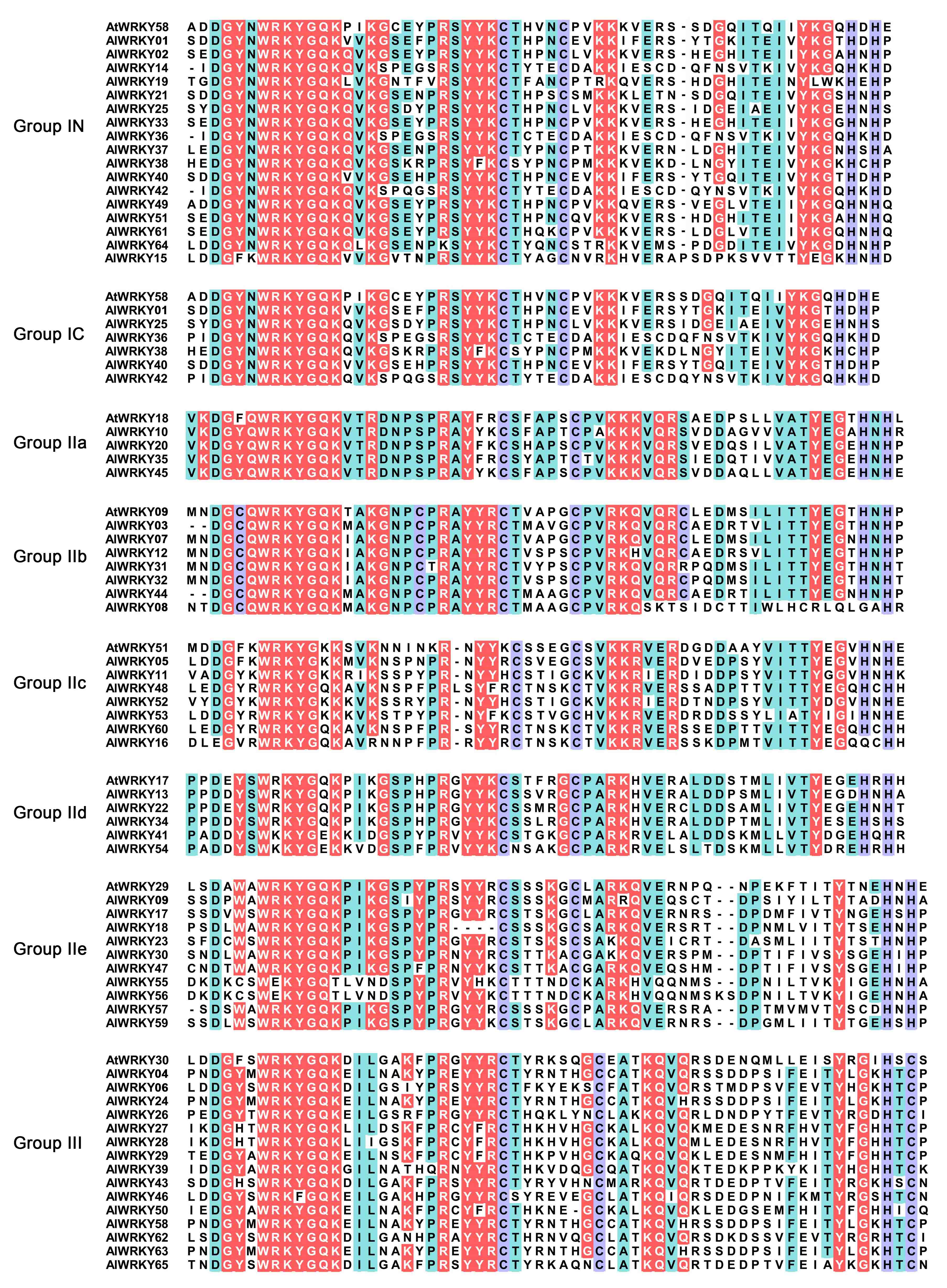
**

**FIGURE S1** Sequence alignment of conserved WRKY domains of AlWRKY transcription factor family proteins.





**FIGURE S2** Chromosomal localization of the AlWRKY gene family.


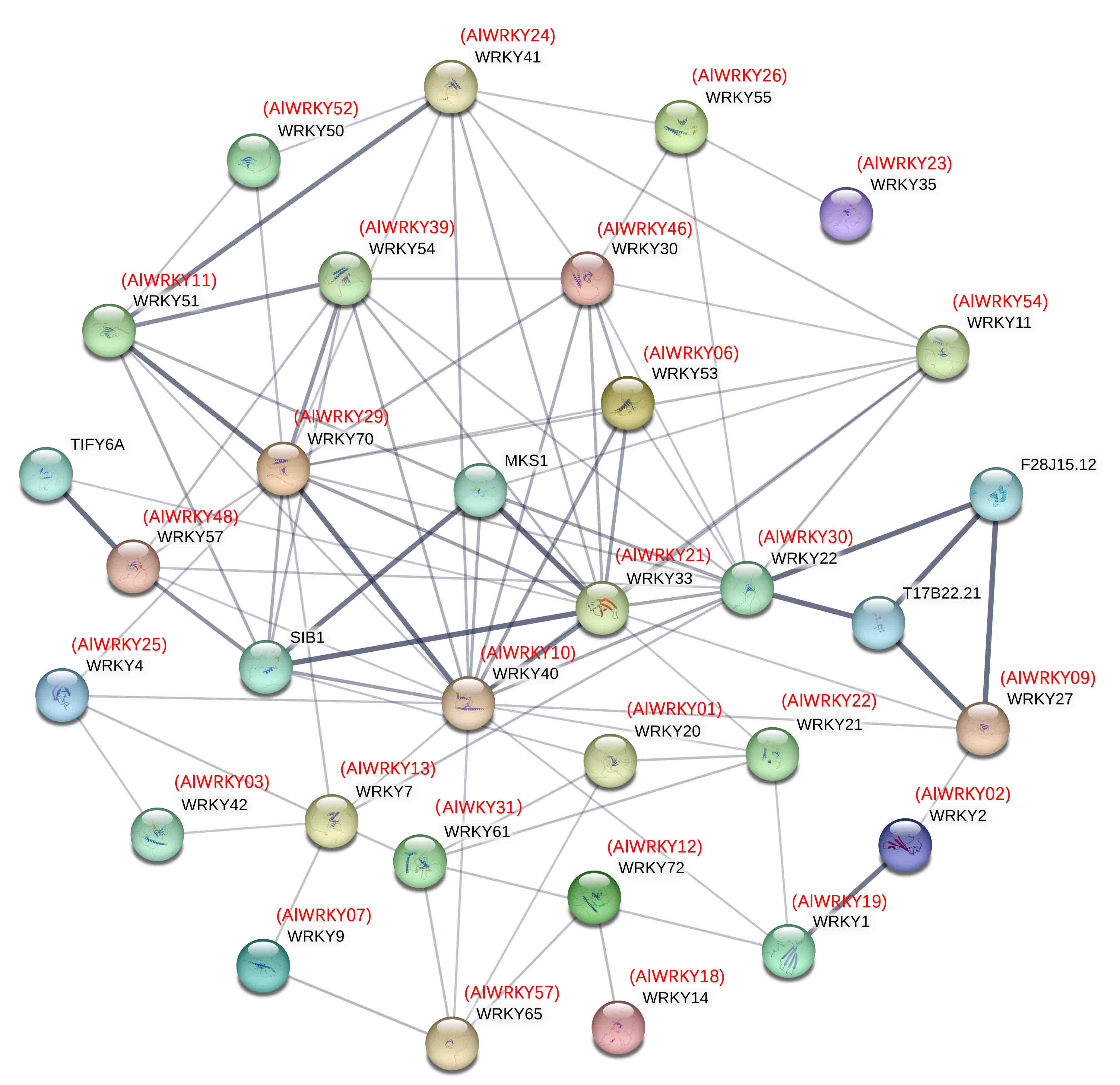


**FIGURE S3** Protein-protein interaction network of specific AlWRKY proteins. Red and black color characters represent *A.lancea* and *A. thaliana*, and the thick lines represent the strength of interaction.


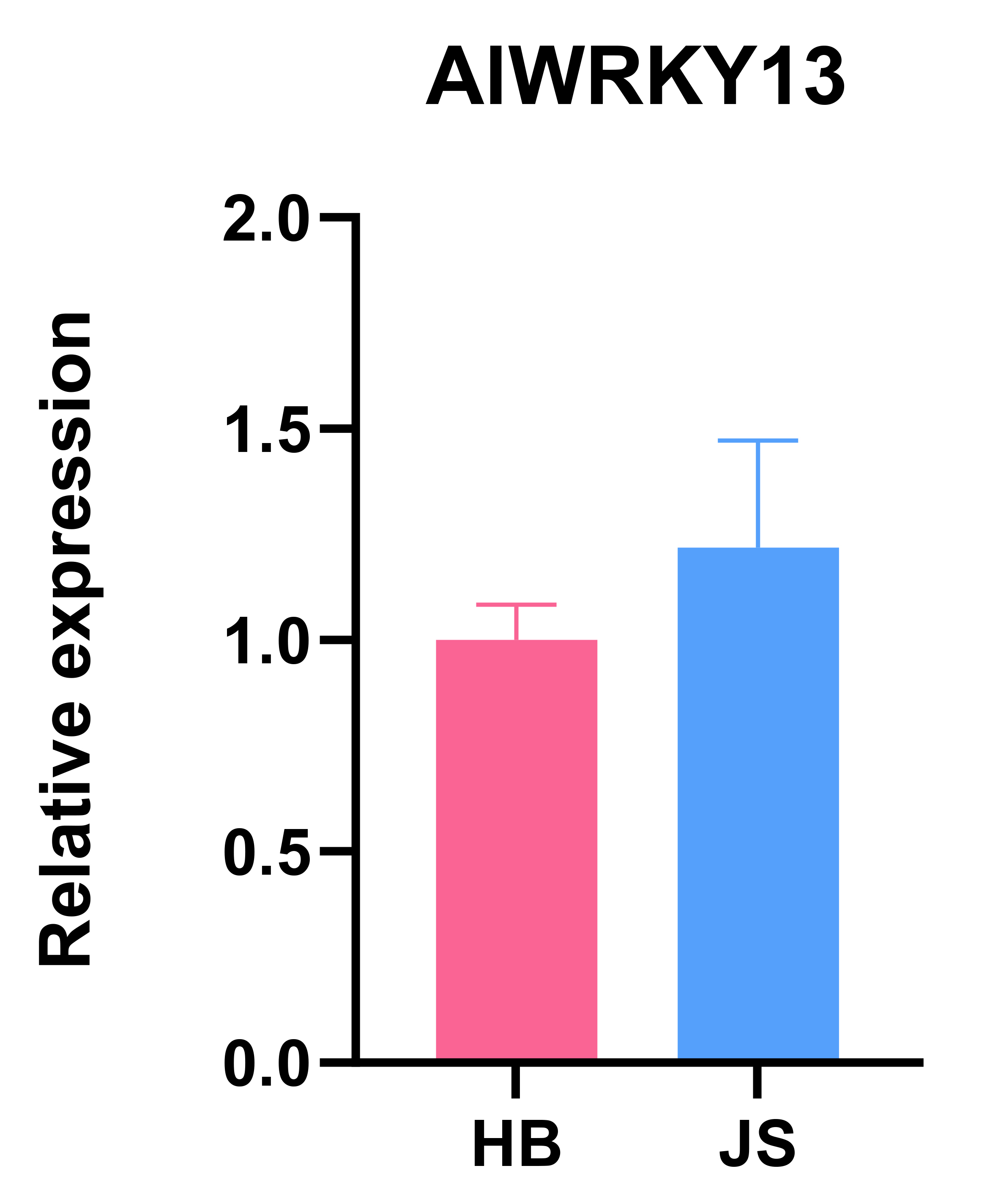


**FIGURE S4** The expression level of AlWRKY13 in *A. lancea* from Hubei and Jiangsu.
